# Changes in corneal endothelial density following scleral buckling surgery for rhegmatogenous retinal detachment: a retrospective cross-sectional study

**DOI:** 10.1186/s12886-018-1015-8

**Published:** 2019-01-05

**Authors:** Chia-Yi Lee, Hung-Ta Chen, Hung-Yu Lin, Hung-Chi Chen, Ling Yeung, Yih-Shiou Hwang, Kuan-Jen Chen, Wei-Chi Wu, Chi-Chun Lai

**Affiliations:** 1 Department of Ophthalmology, Show Chwan Memorial Hospital, Changhua, Taiwan; 2 Department of Optometry, College of Medicine and Life Science, Chung Hwa University of Medical Technology, Tainan, Taiwan; 3 Department of Internal Medicine, Taipei City Hospital- Heping Branch, Taipei, Taiwan; 4 Institute of Medicine, Chung Shan Medical University, Taichung, Taiwan; 5 Department of Optometry, Chung Shan Medical University, Taichung, Taiwan; 6 Department of Optometry, Yuanpei University of Medical Technology, Hsinchu, Taiwan; 7 Department of Exercise and Health Promotion, Chung Chou University of Science and Technology, Changhua, Taiwan; 8 Department of Ophthalmology, Chang Gung Memorial Hospital, Linkou, Taiwan; 9 Center for Tissue Engineering, Chang Gung Memorial Hospital, Linkou, Taiwan; 10 Department of Medicine, Chang Gung University College of Medicine, Taoyuan, Taiwan; 11 Department of Ophthalmology, Keelung Chang Gung Memorial Hospital, Keelung, Taiwan; 12 Department of Ophthalmology, Chang Gung Memorial Hospital, 5 Fuxing Street, Guishan District, Taoyuan, 33305 Taiwan

**Keywords:** Scleral buckling, Human corneal endothelial cells, Cornea, Endothelial cell density, Morphology

## Abstract

**Background:**

To investigate the effect of scleral buckling (SB) on the morphology and density of human corneal endothelial cells (HCEC).

**Methods:**

In this retrospective cross-sectional study, 26 patients who had undergone SB due to rhegmatogenous retinal detachment were enrolled, in which 15 patients received encircling while the other 11 segment types of SB. The postoperative status of affected eye, preoperative status of affected eye, and the contralateral healthy eye was served as the study, control and contralateral groups. The images of the corneal endothelium was obtained by specular microscopy at least three months postoperatively and analyzed.

**Results:**

Postoperative best-corrected visual acuity of the study group was worse than that of another two groups (*P* < 0.001) while intraocular pressure and biometry data were similar. The mean cell area and standard deviation were larger in the study group while the coefficient of variation revealed no difference. The study group manifested a lower endothelial cell density than that of the control and the contralateral (*P* < 0.001) groups. Concerning the percentage of hexagonal cells, the study group showed a lower hexagonality than the control group (*P* = 0.04). No difference of the endothelial morphology was found between the segmental subgroup and the encircling subgroup, nor was a significant difference about endothelial cell loss found in the study group with different measurement interval.

**Conclusions:**

Scleral buckling leads to short-term decreased endothelial cell density and hexagonality, while the rest of morphological features remain unchanged. Moreover, both the segmental and encircling SB procedures yield similar postoperative HCEC status.

## Introduction

Rhegmatogenous retinal detachment (RRD) is generally treated surgically with scleral buckling (SB) with satisfactory attachment rate [1, 2] while visual outcomes after SB are affected by factors such as macular involvement [3] and preoperative visual acuity [4]. Several complications may occur in the posterior segment after SB procedures, such as postoperative abscess formation, extrusion of buckling materials, cystoid macular edema, proliferative vitreoretinopathy, recurrent retinal detachment and choroidal detachment [5]. Concerning the anterior segment, the narrowing of iridocorneal angle may develop after SB [5, 6] and the alterations of corneal curvature, sensitivity and refractive status have also been reported [5, 7].

The corneal endothelium is a monolayer tissue that transports fluid and electrolytes from the corneal stroma and thus maintains the corneal transparency [8]. The morphology of human corneal endothelial cells (HCEC) are different among ethnicities with higher density in Asian population and young age [9, 10]. Due to embryological lineage of the neural crest, damages to HCEC usually lead to permanent endothelial cell loss or corneal decompensation [8, 11]. Among anterior segment procedures that tend to impair HCEC, trabeculectomy, [12] penetrating keratoplasty, [13] phacoemusification [14, 15] and anterior chamber gas endotamponade using sulfur hexafluoride (SF6) [16, 17] would cause a decreased endothelial cell density (ECD) while laser in situ keratomileusis and phacoemusification causes a reduced ratio of hexagonal cells [18, 19].

In the field of vitreoretinal surgeries, vitrectomy [20] and silicone oil injection [21] may result in loss of endothelial cells. Previously, a mean endothelial loss of 12.6% was found in aphakic eyes that received either vitrectomy or SB and the endothelial loss could rise to 16.9% if combined with fluid-gas exchange [20]. Recently, change of ECD was found to be minimal after various retinal detachment surgeries including SB and vitrectomy together [22]. Nevertheless, the sole effect of SB on HCEC remains not fully elucidated because only one study revealed a non-significant decrement of HCEC after SB which did not separate the two subtypes of SB, i.e. encircling and segmental intervention, within the analyses [23].

The aim of this study was to investigate the influence of SB on HCEC both morphologically and quantitatively. In addition, the endothelial morphology in different subtypes of SB were also evaluated.

## Material and method

### Ethnic declaration and subjects

The study adhered to the declaration of Helsinki and was approved by the Institutional Review Board at the Chang Gung Memorial Hospital, Linkou, Taiwan. A retrospective non-randomized cross-sectional study was conducted by chart review and data was collected at the outpatient department. The inclusion criteria of the study group included (1) patients received SB due to RRD and postoperative follow up for more than three months, (2) a corneal endothelial cell examination was available three month preoperatively and postoperatively, and (3) the preoperative corneal endothelium examination was prior to the RRD event. In contrast, the exclusion criteria included (1) patients who received any ocular surgeries before the study (cataract surgery, vitrectomy, pneumatic retinopexy, etc) except intravitreal injection, (2) individual that unable to perform the specular microscope examination, (3) preoperative astigmatism more than 2.00 diopters, (4) patients who received the specular microscope examination more than 30 months postoperatively, and (5) patient with previous diagnosed ocular disease in the contralateral eye. The preoperative status and the contralateral eyes without previous ocular diseases of the included patients were also enrolled for comparison. As a result, there were three groups in the current study: the ophthalmic data from eyes with RRD measured after the SB surgery were regarded as the study group, the ophthalmic data from eyes with RRD measured before the SB surgery were regarded as the control group, and the ophthalmic data from the contralateral healthy eyes measured after the SB surgery were regarded as the contralateral group.

### Surgical procedure

The SB procedures were performed by six experienced vitreoretinal specialists (Lin HY, Yeung L, Hwang YS, Chen KJ, Wu WC, and Lai CC) using standard techniques [24]. Briefly, the surgical steps included cryopexy and meticulous localization of breaks using scleral indentation and binocular indirect ophthalmoscope. A segmental or circumferential silicone sponge buckle (506 style) was then sutured with the matrix 5–0 Dacron sutures. In some patients, trans-scleral drainage of subretinal fluid was performed by cut-down drainage technique using a 25-gauge needle after the choroid was disclosed by a 2.0-mm-long scleral dissection. Room air, sulfur hexafluoride (SF6) or octafluoropropane (C3F8) was injected into the vitreous cavity if necessary. No prominent intraoperative complications were observed and the postoperative care followed the same protocol in all patients.

### Ophthalmic examinations

All the ophthalmic examinations were performed in both eyes. Best-corrected visual acuity (BCVA) was measured by Snellen chart which converted to logMAR for the analysis. Intraocular pressure (IOP) and biometry including spherical equivalent, corneal curvature, central corneal thickness and axial length were collected via Tono-Pen II XL (Medtronic, Jacksonville, FL), autorefractometer (KR-7000, Tokyo, Japan), and A-scan biometry (Echoscan US-800; Nidek, Tokyo, Japan). After the above ophthalmic examinations, we used the non-contact in vivo specular microscope (CEM-530, Nidek, Gamagori, Japan) with nominal magnification of 400X to take photo of the corneal endothelium for evaluation. The technician measured toward the central cornea for one time, and extra attempts would be done if the image quality could not reach the required threshold. After measurements, data was transmitted to the built-in software program provided by the manufacturer then mean cell area, standard deviation, coefficient of variation, ECD and the percentage of hexagonal cell were calculated. All the eyes in the study group, the control group and the contralateral group received identical examinations. If a patient had a BCVA worse than 0.01 and recorded as semiquantitative scale, the value of LogMAR would be set at 1.85 for counting fingers, 2.3 for hand motion, 2.6 for light perception and 2.9 for no light perception according to the experience of Holladay and the University of Freiburg study group results [25, 26]. In addition, the HCEC loss is defined as the preoperative ECD numbers (the control group) minus the postoperative ECD numbers (the study group), and the percentage of HCEC loss was also calculated.

### Statistical analysis

SPSS 20 (SPSS Inc. Chicago, Illinois) was applied for the statistical analysis in the current study. The independent t test was used for the inter-group analysis and the Wilcoxon signed-rank test was applied for the subgroup analysis in the study group according to the type of SB. In the next step, the study group as well as the control group were further divided into another four subgroups according to the interval between the SB surgery and the postoperative HCEC measurement: from three months to six months, from six months to 12 months, from 12 months to 18 months and more than 18 months. Then the HCEC loss, which derived from the difference numbers of ECD between the study group to the control group, in the four subgroups was analyzed using the Kruskal-Wallis test. What should be paid attention to is that we only compared the value between the study group to the control group, and the study group to the contralateral group separately if all the three groups were enrolled in an analysis. Accordingly, an one-way analysis of variance with post-hoc exam for such situation is not necessary. Moreover, the Pearson correction coefficient was used to evaluate the relation between BCVA and change of HCEC morphology, and the correlation between interval of postoperative specular microscope measurement and HCEC loss. To clarify, the HCEC loss was also derived from the difference numbers of ECD between the study group and the control group. A *P* value of 0.05 or less was regarded as significant difference using two-tails probability at 95% confidence intervals. Furthermore, a *P* value less than 0.001 was depicted as *P* < 0.001. The statistic power reached 0.79 under the 0.05 alpha value and medium effect size using G*power version 3.1.9.2 (Heinrich-Heine-Universität, Düsseldorf, Germany).

## Result

There were 26 patients, diagnosed with RRD followed by SB, recruited with mean age of 45.05 ± 14.32 years and mean follow-up period of 9.6 ± 2.1 months which ranged from three months to 26 months after exclusion. The details of basic characters in the study population are shown in Table 1.

**Table 1:** Basic characters of the study population

	Study population
Age (year, mean ± SD)	45.05 ± 14.32
Gender (male to female)	14 to 12
Laterality (right to left)	19 to 7
Type of SB	
Segmental Encircling	11
	15
Gas tamponade	
SF6 C3F8 Room air Not perform	5
	5
	6
	10
Time interval (month, mean ± SD)	
Preoperative HCEC exam to SB SB to postoperative HCEC exam	3.3 ± 1.6
	9.6 ± 2.1

*SD* standard deviation

*SB* scleral buckling

*HCEC* human corneal endothelial cells

After SB procedure, the BCVA of the study group had significantly deteriorated compared to both the control group and the contralateral group (both *P* < 0.001) while the IOP and spherical equivalent of the study group showed similar value to the control group and the contralateral group (*P* > 0.05, respectively). The ophthalmic biometry parameters, including the corneal curvature, central corneal thickness and axial length, also revealed no difference between the two groups (*P* > 0.05, respectively). The comparisons of ophthalmic parameters are shown in Table 2.

**Table 2:** Comparison of ophthalmic parameters between the study and the control groups

Ophthalmic parameters	Study group	Control group	*P1* Value	Contralateral group	*P2* value
BCVA (LogMAR, mean ± SD)	0.69 ± 0.71	0.15 ± 0.31	< 0.001*	0.12 ± 0.22	< 0.001^a^
Intraocular pressure (mean ± SD)	15.02 ± 3.36	13.85 ± 4.66	0.17	13.97 ± 3.08	0.69
Spherical equivalent (mean ± SD)	−4.43 ± 4.72	−3.33 ± 3.91	0.06	−4.33 ± 5.01	0.91
Corneal curvature (mean ± SD)	43.21 ± 1.69	42.67 ± 1.54	0.47	44.25 ± 1.41	0.54
Central corneal thickness (mean ± SD)	552 ± 26	553 ± 24	0.91	550 ± 34	0.79
Axial length (mean ± SD)	25.73 ± 1.54	24.85 ± 1.60	0.07	25.02 ± 1.83	0.40

*BCVA* best-corrected visual acuity

*SD* standard deviation

P1 = The comparison between the preoperative and the postoperative status of the eye received SB

P2 = The comparison between the postoperative status of the eye received SB and the contralateral eye at the same time

^a^denotes significant difference

Concerning the HCEC change, the average cell area was higher in the study group than that in both the control and the contralateral (both *P* = 0.004) groups with a significantly larger standard deviation (*P* = 0.04 and 0.03, respectively). However, the coefficient of variation, which represents the degree of polymegathism, demonstrated no difference between the study and both the control and the contralateral (*P* > 0.05) groups. In contrast, the ECD was significantly lower in the study group than that in both the control and the contralateral groups (both *P* < 0.001) with an HCEC loss of 407 by 14.46% loss respectively. Besides, the percentage of hexagonal cells which indicated pleomorphism showed significant decrement between the study and the control (*P* = 0.04) groups while no significant difference between the study group and the contralateral (*P* = 0.19) groups was found. The comparisons about endothelial morphology between these groups are shown in Table 3. In the subgroup analysis of the study group for the types of SB, no difference regarding the cell area, ECD, pleomorphism or polymegathism was observed between the segmental and encircling subgroups (*P* > 0.05), which is revealed in Table 4. And in another subgroup analysis according to the surgery-to-examination interval, similar HCEC loss was found in all the subgroups despite the different intervals (402 by 14.25% loss, 406 by 14.39% loss, 414 by 14.67% loss and 419 by 14.82% loss, respectively) (*P* = 0.13). The relevant data of HCEC loss are illustrated in Table 5.

**Table 3:** Change of endothelial morphology between the study and the control groups

Index	Study group	Control group	*P1* value	Contralateral group	*P2* value
Average cell area (μm^2^, mean ± SD)	415.67 ± 77.36	371.97 ± 55.83	0.004^a^	374.91 ± 54.23	0.004^a^
Standard deviation (μm^2^, mean ± SD)	166.94 ± 52.27	146.25 ± 47.50	0.04^a^	142.42 ± 43.08	0.03^a^
Coefficient of variation (%,mean ± SD)	39.48 ± 7.27	38.31 ± 7.04	0.56	37.54 ± 7.13	0.35
Endothelial cell density (cell/mm^2^, mean ± SD)	2414 ± 373	2821 ± 255	< 0.001^a^	2796 ± 241	< 0.001^a^
Hexagonality (%,mean ± SD)	45.89 ± 9.69	50.48 ± 7.73	0.04^a^	48.95 ± 8.47	0.19

*SD* standard deviation

P1 = The comparison between the preoperative and the postoperative status of the eye received SB

P2 = The comparison between the postoperative status of the eye received SB and the contralateral eye at the same time

^a^denotes significant difference

**Table 4:** Change of endothelial morphology between the segmental and the encircling subgroups

Index	Segmental subgroup	Encircling subgroup	*P* value
Average cell area (μm^2^, mean ± SD)	411.95 ± 64.81	417.33 ± 90.36	0.91
Standard deviation (μm^2^, mean ± SD)	178.01 ± 60.19	158.57 ± 42.68	0.97
Coefficient of variation (%,mean ± SD)	41.25 ± 9.34	38.31 ± 5.67	0.30
Endothelial cell density (cell/mm^2^, mean ± SD)	2412 ± 328	2415 ± 440	0.79
Hexagonality (%,mean ± SD)	46.70 ± 12.66	45.28 ± 8.42	0.54

*SD* standard deviation

**Table 5:** The reduction rate of corneal endothelial cell loss in the study group by time interval

Time interval (months)^*^	Patient numbers	HCEC loss (mean ± SD)	Reduction rate (%)	*P* value
3 to 6	5	402 ± 40	14.25	
6 to 12	11	406 ± 45	14.39	
12 to 18	6	414 ± 47	14.67	
> 18	4	419 ± 46	14.82	
				0.13

*HCEC* human corneal endothelial cells

*SD* standard deviation

^*^The time interval is defined as the period from the scleral buckle surgery to the postoperative human corneal endothelial cell examination

**Fig. 1:**
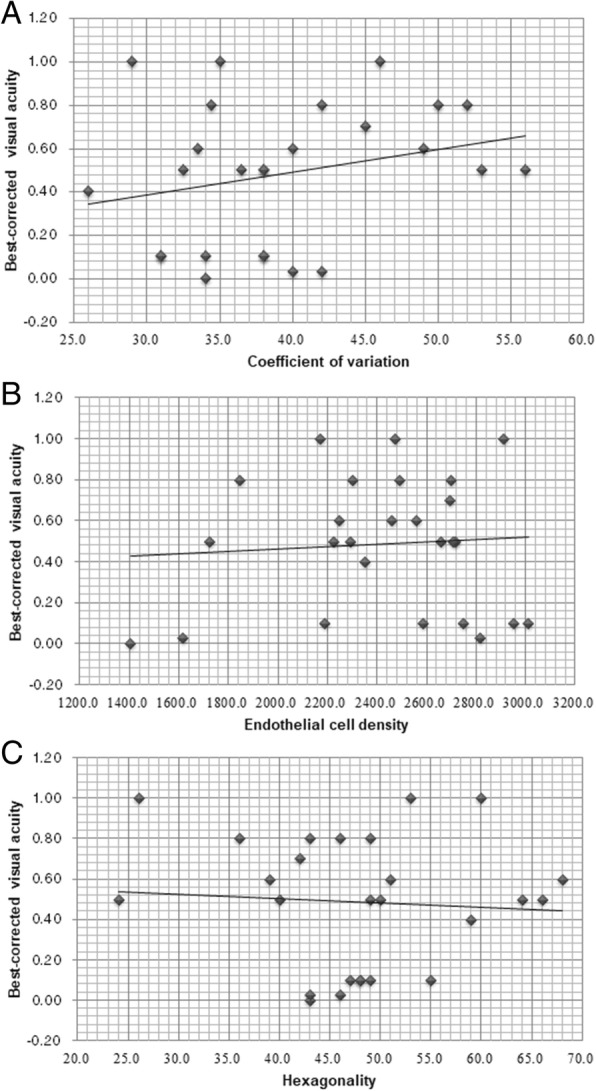
The linear correlations between visual acuity and corneal endothelial morphology including coefficient of variation (**a**), endothelial cell density (**b**), and hexagonality (**c**)

**Fig. 2:**
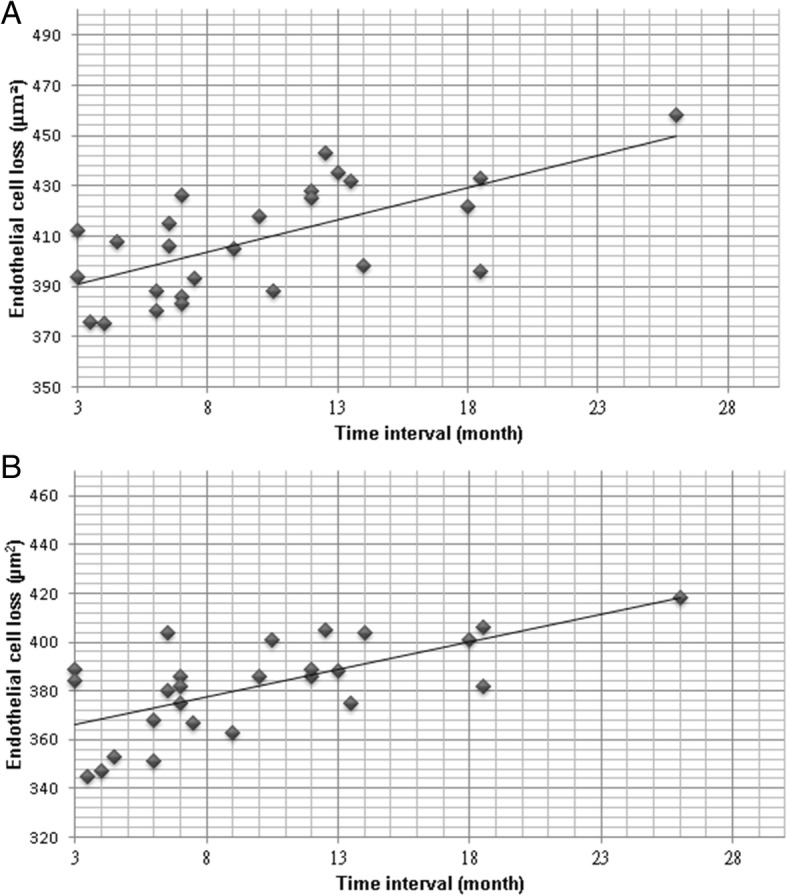
The linear correlations between the human corneal endothelial cell loss and the postoperative examination duration including the correlation between the study and the control group (**a**) and the correlation between the study and the contralateral groups (**b**)

For the correlation of BCVA to other parameters, Pearson correlation analysis illustrated the positive correlations between the BCVA and polymegathism (*r* = 0.26), ECD (*r* = 0.01) and pleomorphism (*r* = − 0.05) in the study group. None of them was significantly correlated (*P* > 0.05) and the scatter diagrams of those indexes are shown in Fig. 1. In addition, the time of HCEC measurement was also positive correlated to the HCEC loss in the study group based on both the control (*r* = 0.37) and the contralateral (*r* = 0.34) groups. Nevertheless, both the correlations are not significant (*P* > 0.05), and the scatter diagrams are demonstrated in Fig. 2.

## Discussion

Briefly, the BCVA in the study group was worse than that in the control group and the contralateral group while other ocular parameters remained similar. About the endothelial morphology, the study group had larger cell area with larger standard deviation than that in the control group and the contralateral group but with similar coefficient of variation. The ECD in the study group was lower after the SB procedure with a decrement of hexagonality compared to that in the control group.

Possible postoperative complications of SB include postoperative astigmatism, myopia shift, axial length elongation and elevated higher order aberrations [27, 28]. In previous reports, SB was reported to reshape the anterior chamber depth and posterior corneal surface [29, 30]. In addition, anterior segment ischemia was reported which account for 3 % in the complications of SB and could even lead to ruberosis iridis [31, 32]. Accordingly, SB owns the chance to weaken HCEC to some degree by both mechanical and circulatory alternations, which may explains the results in the current study. Specifically, we speculate that the effect of SB to HCEC is an acute damage rather than a chronic influence. In previous reports that evaluated the alteration of anterior segment after retinal surgery including SB, the change of anterior segment were mostly observed within one year [23, 27, 29]. In addition, the anterior chamber depth and central cornea thickness had no correlation to the interval after SB in the study written by Huang et al [27]. Moreover, the anterior segment ischemia is an early postoperative complication that can remit within weeks, [33] thus the circulation defect which may damage HCEC is probably due to an acute distress. The average HCEC loss was similar among subgroups with different surgery-to–examination interval in the current study, which also supported this concept.

In the current study, a significant HCEC loss of 407 by 14.46% loss (*P* < 0.001) was found in the study group compared to that in the control group and the ECD number was also significant difference between the study group and the contralateral group, which was contradictory to what was reported recently [23]. Moreover, the significantly enlarged cell area in the study group further demonstrated that the quantity of HCEC was impaired after the SB. The decrement of ECD concerning percentage is similar to the HCEC loss in cataract and corneal transplantation surgeries with normal ECD and a follow up period from one month to 20 years, which might lead to corneal decompensation in some cases [34–37]. A possible explanation for the conflicting outcomes between the current study and the other by Mukhtar et al. is that the latter excluded various common ocular disorders which might damage the corneal endothelium [23]. The average ECD in the study group was above 2400/mm^2^ thus the development of corneal decompensation in our patients was less likely. Nevertheless, whether SB would result in corneal decompensation in patients with lower ECD is uncertain. On the other hand, the different subtypes of SB may not influence the postoperative HCEC status since no difference concerning the HCEC parameters was found between the segmental and encircling subgroup in the current study, which was not reported elsewhere.

Recently, several studies demonstrated progressive decrement of HCEC after different surgeries such as cataract surgery, penetrating keratoplasty and Descemet’s stripping automated endothelial keratoplasty [38–40]. For the possible mechanism, the alteration of the intraocular microenvironment might lead to the HCEC loss with elevation of cytokine including interleukin, monocyte chemotactic protein and interferon [39–41]. In the current study, although the highest HCEC reduction rate occurred in those with longest surgery-to-examination interval, the results did not show any significant difference among the four subgroups. Furthermore, the correlation analysis also revealed a positive but non-significant correlation between the HCEC loss and the surgery-to-examination interval. Accordingly, the intraocular microenvironment might not be altered prominently after SB.

About other morphological indexes of HCEC, the similar coefficient of variation of the study group compared to both the control group and contralateral group in the current study indicates that the polymegathism of HCEC does not change after the procedure of SB. On the other hand, the percentage of hexagonal cells changed significantly only between the study and the control groups (*P* = 0.04). Since the HCEC endothelial status in one eye are similar to the fellow eye in Chinese population, [42] the results should be similar while comparing the study group to these two groups. Still, the percentage of hexagonal cells in the control group was slightly higher than the contralateral group which might own an advantage in statistical analysis.

The impaired HCEC may lead to corneal edema which produces light scattering thus threatening the visual outcome [8, 9]. Fortunately, there was not corneal edema detected in our patients. As a result, the significantly worse BCVA in the study group may be associated with other reasons such as retinal corruption resulting from RRD or myopic change due to increased corneal curvature [7] than the damage on the corneal endothelium. For the rest of ophthalmic parameters, there was no difference between the study group to the rest two groups except for a silently longer axial length in the study group, which might not have profound effects on the visual acuity.

Concerning the surgery that may influence the HCEC, cataract surgery with phacoemulsification may cause HCEC loss [18] which would exacerbate in patients with type 2 diabetes mellitus [37] and triple procedure may be demanded in some patients to restore visual acuity [43]. Other surgeries that contribute to prominent endothelial damage include penetrating keratoplasty, laser in situ keratomileusis, Ahmed valve implantation, mitomycin C-augmented trabeculectomy and trans pars plana vitrectomy which mainly alter either the ECD or pleomorphism [12, 19, 20, 22, 36, 44]. Similar to those intraocular interventions mentioned above, SB also showed negative effects on HCEC in our study. In another prospective study published recently, the dexamethasone intravitreal implant also lead to the decrement of ECD in patient with retinal venous occlusion [45]. Accordingly, the use of corticosteroid in patient received SB should be caution to prevent double injury to the HCEC.

There are still some limitations in our study. First, the small study population and unequal follow-up period may contribute to some bias. In addition, only one specular microscopic examination was done postoperatively in the study population due to the retrospective design, thus the reduction rate of each patient could not be accessed. Besides, the interval of preoperative HCEC measurement was also different which may influence the accuracy. However, the interval of preoperative examination was relative similar which ranged from six weeks to six months thus the influence may be minor compared to the postoperative condition.

## Conclusion

In conclusion, the SB may lead to short-term decline of ECD and pleomorphism while the polymegathism remains unchanged. In addition, the different subtypes of SB, whether segmental or encircling approach, may yield similar postoperative HCEC status. Still, further large-scale studies with a longer follow-up period to investigate the effect of SB on patients with low endothelial count to find the lower threshold of SB in such population are mandatory.

## Abbreviations

ECD: Endothelial cell density
HCEC: Human corneal endothelial cells
RRD: Rhegmatogenous retinal detachment
SB: Scleral buckling
